# Spectrum of primary immunodeficiency disorders in Sri Lanka

**DOI:** 10.1186/1710-1492-9-50

**Published:** 2013-12-27

**Authors:** Nilhan Rajiva de Silva, Sepali Gunawardena, Damayanthi Rathnayake, Geethani Devika Wickramasingha

**Affiliations:** 1 Department of Immunology, Medical Research Institute, Colombo 08, Sri Lanka

**Keywords:** Primary immunodeficiency, Common variable immune deficiency, X linked agammaglobulinemia, Severe combined immune deficiency

## Abstract

**Background:**

While primary immunodeficiencies (PID has been recognized in the west for decades, recognition has been delayed in the third world. This study attempts to detail the spectrum of PID, the therapy provided, and constraints in the diagnosis and treatment in a middle income country such as Sri Lanka.

**Methods:**

Nine hundred and forty two patients with recurrent infections and features suggestive of immune deficiency, referred from the entire country in a 4 year period, to the sole immunology unit in Sri Lanka were included. The following tests were performed. Full blood counts, serum Immunoglobulin and complement C3 and C4 levels, functional antibody levels, enumeration of lymphocyte subsets, in vitro and in vivo T cell functional assays,, nitroblue tetrazolium assay to diagnose chronic granulomatous disease, hair shaft assay to diagnose Griscelli syndrome. Sequencing of the common gamma chain to identify x linked severe combined immune deficiency, and X linked agammaglobulinemia was confirmed by assaying for Btk mutations by single sequence conformation polymorphism. HIV/AIDS was excluded in all patients.

**Results:**

Seventy three patients were diagnosed with a primary immune deficiency. The majority (60.27%) had antibody deficiency. Common variable immune deficiency was the commonest (28.76%), followed by X linked agammaglobulinemia (XLA) (20.54%). Five patients had possible hyper IgM syndrome.

Ten patients had severe combined immune deficiency (SCID), including 2 with x linked SCID, in addition to DiGeorge syndrome (2), ataxia telangiectasia (6), autosomal dominant hyper IgE syndrome (2), chronic granulomatous disease (4), leucocyte adhesion deficiency type 1 (2) and Griscelli syndrome (3).

Patients with autoinflammatory, innate immune and complement defects could not be identified due to lack of facilities.

**Conclusions:**

Antibody deficiency is the commonest PID, as in the west.IgA deficiency is rare. Autoinflammatory diseases, innate immune and complement deficiencies could not be identified due to lack of diagnostic facilities. Lack of awareness of PID among adult physicians result in delay in treatment of adult patients. While treatment of antibody deficiencies provided in state hospitals has extended life expectancy, there is no treatment available for severe T cell defects.

## Background

Primary Immunodeficiencies (PID) are monogenic diseases, and around 150 such diseases have been identified [1]. Many are yet to be identified. A prevalence of 1: 2000 is reported in the US, even though this may be an under estimate [2]. The figures may differ in other countries, for example Brazil etc. Patients with PID present with recurrent infections, but may also have autoimmune diseases, allergy and malignancy [3].

The International Union of Immunological societies (IUIS) have classified PID under 8 groups, including humoral, combined, phagocytic, innate immune and complement defects [4]. In the west, PID has been diagnosed for more than half a century, whereas in the third world, recognition of PID is only a few decades old. Diagnostic and research facilities are few, and therapy is delayed or not available. Delay in the diagnosis of x linked agammaglobulinemia till 10 years of age, for example, raises the risk of chronic lung disease to 40% [5].

Sri Lanka is a recent middle income country (per capita GDP US $ 2000). It has very few trained immunologists, and only one dedicated Immunology unit, at the Medical Research Institute, Colombo. Patients with recurrent infections and suspected immunodeficiency are referred from the entire country to this unit. The patients are diagnosed and referred back with treatment recommendations.

This study attempts to detail the spectrum of PID identified in a 4 year period from 2008 to June 2012, the therapy provided, and constraints in the diagnosis and treatment in a middle income country such as Sri Lanka.

## Material and methods

Nine hundred and forty two patients with recurrent infections and features suggestive of an underlying immune deficiency [6] (Table 1), referred to the Department of Immunology, Medical Research Institute Colombo, Sri Lanka were investigated for underlying primary immunodeficiency (PID). These patients were referred to the department, from all regions in Sri Lanka as it is the only dedicated Immunology unit in the country. A total of 10 ml of blood was taken for investigations and the following tests were done; the complete blood count, flowcytometry (to quantify T lymphocytes and subsets (CD 3, CD 4 and CD 8), B lymphocytes (CD 19), and Natural Killer cells (CD 16/56)(Epics XL, Beckmann Coulter [7], the monoclonal antibodies were from Becton Dickinson, BD), serum immunoglobulin (IgG, IgA, IgM) levels by radial immune diffusion (RID) [8]^,^, serum IgE by ELISA (Roche), serum Complement C3 and C4 (RID) [9], and isohemagglutinin levels. T lymphocyte proliferation was performed, using the mitogen concanavalin A [10]. A Mantoux test, using purified protein derivative (PPD RT 23) 2TU 0.1 ml given intra dermally, with the diameter of the induration read at 72 hours, was performed as an in vivo test of T lymphocyte function. An induration diameter >0 mm was read as a positive delayed type hypersensitivity reaction [11]. The nitro blue tetrazolium assay (NBT) was performed on freshly collected blood samples [12] and mutations in Bruton’s tyrosine kinase (Btk)were identified by single sequence conformation polymorphism (SSCP) [13]. HIV/AIDS was excluded in all patients using Genscreen™ ULTRA HIV Ag-Ab ELISA to detect the P24 antigen and antibody to HIV 1 and 2.

**Table 1:** **Clinical features suggestive of Immunodeficiency**[6]

**History**	**Number (%)**
	**(n = 942)**
**Age**	
Birth - < 1 year	362 (38.4)
1 - < 5 years	359 (38.15)
5 - < 12 years	49 (5.2)
12- < 18 years	41 (4.35)
18- < 30 years	49 (5.2)
> 30 years	82 (8.7)
**Sex**	
Male	526 (55.8)
Female	416 (44.16)
**Recurrent (proven) infection**	
≥ 2 severe infections (pneumonia, sepsis, meningitis, osteomyelitis)	393 (42.7)
Atypical presentation of infection	6 (0.6)
Unusually severe course, or impaired response to treatment	230 (24.4)
Unusual or opportunistic agent	10 (1.0)
Recurrent infections with same type of pathogen	60 (6.3)
Abscesses of internal organs, or recurrent subcutaneous abscesses	115 (12.2)
FTT with prolonged/recurrent diarrhoea	24 (2.5)
Generalized long lasting warts or molluscum contagiosum	5 (0.5)
Extensive prolonged candidiasis	16 (1.7)
Delayed separation (> 4 weeks) of umbilical cord	2 (0.2)
Delayed shedding of primary teeth	2 (0.2)
F/H of infant deaths, ID, consanguinity	16 (1.7)
Difficult to treat obstructive lung disease, unexplained bronchiectasis	50 (5.3)
**Physical examination**	
Dysmorphic features, especially facial abnormalities, microcephaly	0
Partial albinism, abnormal hair, severe eczema	3 (0.3)
Telangiectasia, ataxia	10 (1.0)
Gingivitis, oral ulcers/aphthae	0
Absence of immunological tissue	0
Organomegaly	0
Digital clubbing	0

The patients were immunized with the typhoid Vi vaccine (Serum Institute, India), and a further blood sample taken 4 weeks later, and the 2 samples were tested for seroconversion using a typhoid Vi ELISA kit (Binding Site. UK) [14].

The specific PID was identified by the clinical features and results of the investigations performed [14]. Griscelli syndrome was diagnosed by examining the hair shaft for clumping of melanin pigment [15], while leucocyte adhesion deficiency was diagnosed by flowcytometry: the granulocytes were evaluated for the presence of CD 18 (BD) [16].

XLA was diagnosed in male patients with agammaglobulinemia, absent B cells and a genetic diagnosis using SSCP [13].

CVID was diagnosed in patients according to guidelines of the European Society of Immunodeficiencies (ESID) and the Pan American Group for Immunodeficiencies [17]. Patients over 2 years of age, with low (< 2 SD) IgG, and a low IgA or IgM, and no seroconversion following immunization with the typhoid Vi vaccine or absent isohemagglutinins and after exclusion of other causes of hypogammaglobulinemia were considered as having CVID. Conditions such as XLA, Good’s syndrome (no thymoma by HRCT), proteinuria, protein losing enteropathy, use of drugs such as Rituximab, anti convulsants etc. were excluded in these patients [18].

Severe combined immune deficiency (SCID) was diagnosed by flowcytometry [19], ataxia telangiectasia (AT) in children by the presence of ataxia, ocular cutaneous telangiectasia and elevated alpha feto protein levels [20] and Di George syndrome by the presence of neonatal hypocalcaemia, conotruncal cardiac defects, typical facies and absent thymus [21]. Autosomal dominant hyper IgE syndrome [22] was diagnosed using the National Institutes of Health (NIH) scoring system, with scores of over 40 suggestive of the diagnosis [23]. Chronic granulomatous disease was confirmed by a positive NBT assay [12].

*Pneumocystis jiroveci* was diagnosed from respiratory secretions and broncho alveolar lavage using the Grocott-Gomori methenamine silver (GMS) stain [24] by a trained mycologist. *Candida guillermondi* was cultured from blood [25].

The study was partly sponsored by the World Health Organization (WHO), as part of study on polio excretion in patients with PID. Ethics approval was granted by the Medical Research Institute, Colombo, Sri Lanka. Written, informed consent was obtained from the patients or parents in the case of children less than 18 years.

## Results

Seventy three patients were diagnosed with a primary immune deficiency (Table 2). Fifty three (72.6%) were ≤ 12 years, 12 (16.4%) ≥18 years and 8 (10.9) ≥ 30 years. The male to female ratio was 1.3: 1. Seven of the 12 patients aged ≥ 18 years, and 5 of 8 aged ≥ 30 years were female. One patient with x linked SCID was diagnosed in utero (20 weeks of pregnancy), and diagnosis confirmed at birth. The majority (60.27%) had antibody deficiency. Common variable immune deficiency was the commonest clinically significant PID (28.76%), followed by X linked agammaglobulinemia (XLA) (20.54%). There were 3 sets of siblings among patients with XLA. Of the 5 patients with hyper IgM syndrome, 3 patients, all male, developed symptoms before the age of 2 years, and had opportunistic infections (2 with *P. jiroveci* pneumonia and one who had *C. guillermondi* cultured in the blood on two occasions). All 3 probably had deficiencies of either CD 154 (CD 40 L), or CD 40. One patient was subsequently identified as having CD 40 deficiency in the US, and successfully underwent stem cell transplantation [26]. One other patient had lymphadenopathy and giant germinal centers, indicating a possible activated cytidine deaminase deficiency [27]. One patient had partial IgA deficiency, but functional antibody levels were not available.

**Table 2:** Spectrum of primary immune deficiency

**Disease**	**Number**	**Sex**		**Age**					
	**(%)**	**M**	**F**						
**Combined**	**10 (13.6)**			**< 1 year**	**1 - < 5 years**	**5 < 12 years**	**12-<18 years**	**18-<30 years**	**≥30 years**
Severe combined immuno deficiency (including X linked = 02 Omenn syndrome = 01)	10 (13.6)	04	06	10	-	-	-	-	-
**Well defined syndromes**	**10 (13.6)**								
Ataxia telangiectasia	06 (8.2)	02	04	-	03	03	-	-	-
Di George syndrome	02 (2.7)	01-	01	02	-	-	-	-	-
Hyper IgE syndrome (autosomal dominant)	02 (2.7)	01	01	-	-	01	01	-	-
**Antibody deficient**	**44 (60.27)**								
X Linked agammaglobulinemia	15 (20.54)	15	0	01	07	05	02	-	-
Autosomal recessive agammaglobulinemia	02 (2.7)	0	02	-	01	01		-	-
Common variable immune deficiency	21 (28.76)	10	11	-	01	04	04	04	08
Partial IgA deficiency	01 (1.36)	0	01	-	-	-	01	-	-
Hyper IgM syndrome (including CD 40 deficiency = 01)	05 (6.8)	03	02	-	02	03	-	-	-
**Immune dysregulation**	**03 (4.1)**								
Griscelli syndrome	03 (4.1)	02	01	03					
**Phagocytic defects**	**06 (8.2)**								
Chronic granulomatous disease	04 (5.4)	04	0	01	02	01	-	-	-
Leucocyte adhesion deficiency type 1	02 (2.7)	0	02	02	-	-	-	-	-
**Autoinflammatory**	**0**	**-**		**-**	**-**	**-**	**-**	**-**	**-**
**Innate immune defects**	**0**	**-**		**-**	**-**	**-**	**-**	**-**	**-**
**Complement defects**	**0**	**-**		**-**	**-**	**-**	**-**	**-**	**-**
**Total**	**73**	**42 31**		**19**	**16**	**18**	**08**	**04**	**08**

Ten patients had severe combined immune deficiency (SCID), including one patient with Omenn syndrome with features of erythroderma, alopecia, hepatosplenomegaly, lymphadenopathy and eosinophilia [19]. Of the other 9 patients with SCID, 5 were T-B + (2 males), and 4, T-B-. The 2 males with T-B + SCID were diagnosed as having x linked SCID. Sequencing of the common γ chain of the IL 2 receptor revealed mutations. One of these patients, with a family history of 15 male infant deaths spanning 3 generations, underwent stem cell transplantation in India, and is 3 years old at the time of writing. Except for two patients with X linked SCID, all others succumbed during infancy.

The patients with DiGeorge syndrome had symptoms of hypocalcaemia, cardiac defects (one patient) and typical facies (Table 3). The immune system however, was not affected. Chromosomal studies could not be done. Two patients were diagnosed with autosomal dominant hyper IgE syndrome, both with National Institutes of Health scores > 40, suggestive of the diagnosis (Table 3). Ataxia telangiectasia was diagnosed in 6 patients, all having ataxia, telangiectasia, low IgA levels and elevated α feto protein.

**Table 3:** Clinical features of Autosomal dominant hyper IgE syndrome, Ataxia telangiectasia, Di George syndrome

**Disease**	**Patient age and sex**	**Clinical features**
**Hyper IgE syndrome**	15 years, male	New born rash, seborrheic dermatitis from 3 months, recurrent lower respiratory infections (> 6), pneumonia (X3), pneumatoceles, oral thrush, recurrent skin abscesses, typical facies, nasal width, fractures (left and right radius and ulna), Eosinophilia (> 800 / μl), IgE > 2000 IUml NIH Score 63*
	9 years, female	New born rash, dermatitis, recurrent skin abscesses (< 4), pneumonia (1 episode), pneumatocele, retained primary teeth, hyperextensibility of joints, recurrent upper respiratory infections, eosinophilia > 800 / μl, IgE > 2000 IU/ml NIH Score 41*
**Ataxia telangiectasia**	9 years, female	Recurrent respiratory infections from 2 1/2 years, squint and bilateral ocular telangiectasia, ataxia
		Bronchiectasis
		No consanguinity
		Alpha feto protein 111.4 ng/ml (< 8 ng/ml)
		Reduced IgA
		CT Brain – prominent lateral and 4^th^ ventricle, no cerebellar atrophy
	3 years, female	Ataxia at 3 years, torticollis, ocular telangiectasia, sister diagnosed with ataxia telangiectasia
		IgG, IgA reduced, increased IgM
	1 ½ years, female	Unsteady gait
		Elder sister died at 11 years with ataxia telangiectasia
		Ocular, ear lobe telangiectasia
		Ataxia +
		IgA reduced
		Alpha feto protein 48.6 ng/ml (< 8 ng/ml)
	8 years, male	Ataxia, intention tremor
		Ocular telangiectasia
		Recurrent respiratory tract infection
		IgA reduced
		Alpha feto protein 156 ng/ml (< 8 ng/ml)
	5 years, female	Walking milestones delayed, ataxia after 1 ½ years, bulbar telangiectasia, oculomotor apraxia, dyskinesia, dystonia
		Immunoglobulin levels normal
		Alpha feto protein 217.9 ng/ml (< 8 ng/ml)
	4 ½ years, male	Imbalance while walking, inability to keep posture at 1 ½ years, with progressive worsening
		2 attacks of lower respiratory infection
		Ocular telangiectasia. Ataxia
		IgA reduced
		Alpha feto protein 144.52 ng/ml (<8 ng/ml)
DiGeorge Syndrome	10 month, male	Recurrent respiratory tract infection from 3 months
		Hypocalcemia (no fits)
		Dysmorphic facies(micrognathia,low set ears, thin upper lip, prominent philtrum, prominent forehead, high arched palate.
		Absent thymus (chest xray, ultra sound scan)
		2 D Echo–normal heart
		Serum immunoglobulins, lymphocyte subsets normal
	3 years, female	Recurrent respiratory infections, dysmorphic facies (micrognathia, prominent philtrum, low set ears)
		Hypocalcemia = serum Ca^++^ 1.14 mmol/l(2.15-2.55)(no fits)
		2D Echo = Tetralogy of Fallots, right sided aortic arch
		Serum immunoglobulins = IgG and IgA low, IgM normal
		Lymphocyte subsets and function normal

*NIH score–National Institutes of Health score [23].

Of the 5 patients with chronic granulomatous disease one was probably x linked, as 50% of the mother’s neutrophils gave abnormal results with the NBT test. Two siblings had leucocyte adhesion deficiency type 1(LAD type 1, with delayed separation of the umbilical cord, necrotic skin ulcers with minimal pus, high neutrophil counts and <1% of gated neutrophils having CD 18 by flowcytometry.

Three patients with Griscelli syndrome were investigated for underlying immune deficiency, and were found to have an intact immune system. Genetic identification of a RAB 27A mutation was not attempted [28].

Three pairs of siblings had XLA, in addition to the siblings with LAD.

Due to lack of diagnostic facilities, patients with auto inflammatory, innate immune and complement defects could not be identified.

## Discussion

Seventy three patients were identified as having a primary immune deficiency. Males were affected more than females (1.3:1), a trend seen in other parts of the world, including Europe [29] and India [30].It was suggested that the higher percentage of males with primary immunodeficiency (PID) in India is at least partly attributed to a bias in seeking better medical care for male children that still exists In that society [30]. While India is Sri Lanka’s closest neighbor, with religious and cultural affinities to Sri Lankans, this is unlikely to be the reason for the male preponderance in our study. However, females predominated ≥18 years, and more so ≥30 years. In the European Society of Immunodeficiency (ESID) registry, more women than men are affected by a PID in patients older than 30 years, the reason being unclear [29].

The majority (60.27%) had an antibody deficiency. This is in keeping with results in Europe [29], Turkey [31], Iran [32] Japan [33] and a center in India [34].Other centers in India report immune dysregulation and B and T cell disorders [30], and immune dysregulation and phagocytic disorders [35] as the commonest PID. However, these centers deal with pediatric patients. Common variable immune deficiency (CVID) was the commonest PID (28.7%) in our study, as in Europe (21.01%)[29] and Iran (20%) [32]. CVID is less common in Japan (11%) [33], and is under diagnosed in India [30,34,35] as the data from India are from pediatric centers. The diagnosis of CVID has increased from 2010 in our center due to the education programs conducted among respiratory physicians regarding PID. In fact, around a fourth of our patients with PID are ≥ 12 years, and under the care of adult physicians. IgA deficiency was seen in only one patient, and that too a partial deficiency. IgA deficiency is the second commonest immune deficiency in the European registry [29] but is uncommon in Japan [33], India [30] and Iran [32]. This may be because most patients with specific IgA deficiency lack clinical manifestations [33].

X linked agammaglobulinemia (XLA) is the second most common PID in our study (20.54%). These figures are similar to Indian studies among mainly pediatric patients, 19.1% [30], and 19.9% [34]. European figures are much less (5.93%) [29]. It is the commonest PID in Japan (14.7%) [33]. However, the percentage is lower as better diagnostic techniques available enable Japan (and European countries) to identify a larger spectrum of PID, unlike in lower resource countries such as India and Sri Lanka.

Of the five patients diagnosed as having hyper IgM syndrome (HIGM), one was found to have a CD40 deficiency [26], which is seen in < 1% of patients with HIGM [27]. A genetic diagnosis is not possible at present in Sri Lanka due to lack of flowcytometric and genetic assays.

Two of the 10 patients with severe combined immune deficiency (SCID) had mutations in the common γ chain and had x linked SCID. Of the 4 patients with T-B + SCID, 2 were females. While the sample size is small, X linked SCID was present in only 20% of patients. While X linked T –B + SCID is reported to more frequent in the rest of the world [13,36], autosomal recessive SCID is more common in countries with increased consanguinity such as Iran [32].

The frequency of phagocytic defects in our study is similar to European figures, with chronic granulomatous disease (CGD) being the commonest. Phagocytic disorders are much more frequent in certain Asian countries. CGD is the second most common PID in Iran [32], where the majority have an autosomal recessive inheritance, and in Korea [36].

Complement defects were not identified as only complement C3 and 4 assays were available, and terminal path deficiencies, particularly in patients with recurrent neisserial infections, could not be confirmed. We could not test for innate immune defects and auto-inflammatory conditions. However, these are rare defects and identified infrequently in the best of centers [29]. While patients with features suggestive of periodic fevers are referred to our unit, lack of supportive tests, including gene sequencing in Sri Lanka precludes confirmation of a diagnosis of periodic fever. This is true for innate immune defects as well.

Tuberculosis and infections with other mycobacterial infections are common in Sri Lanka. Patients with disseminated mycobacterial (due to *M.tuberculosis* or non tuberculous mycobacteria) and recurrent drug sensitive tuberculosis in treatment compliant patients are diagnosed. A patient with Mendelian susceptibility to mycobacterial disease (IL 12 R B1 deficiency) has been reported in Sri Lanka [37], but laboratory confirmation had to be carried out in the UK due to lack of diagnostic facilities.

As in other less developed countries [34], there are many challenges to be overcome in the management of PID.

Most patients referred to our unit are from Pediatric units. Lack of knowledge among physicians as opposed to pediatricians, regarding primary immune deficiency is responsible for the delay in diagnosis of the many adult patients with CVID. In addition, the number of trained Immunologists is inadequate to deal with all the patients referred for immunological evaluation (including allergy). Education of registrars in internal medicine and specialties such as respiratory medicine, and General practitioners is ongoing.

Patients with features suggestive of immunodeficiency should be investigated. These features include [38] severe, potentially life threatening infections, persistent infections despite adequate treatment, recurrent infections inappropriate for age, and infections with poorly pathogenic organisms. In addition, failure to thrive in infancy, lymphopenia in infancy and combination of features characteristic of immunodeficiency syndromes (eg, ataxia telangiectasia) should indicate need for further evaluation. A family history of immune deficiency was a significant feature in the identification of PID in our sample. Secondary immunodeficiencies (including HIV/AIDS) should be excluded in all cases. As diagnostic facilities are limited in our setting investigations should be targeted according to the possible underlying PID [14,38] (Table 4). This will minimize costs and improve diagnostic services.

**Table 4:** Investigation of PID

**PID**	**Clinical features**	**Suggested investigation**
Combined (T and B cell deficiency)	Failure to thrive, severe viral, intracellular bacterial (atypical, extrathoracic or disseminated tuberculosis, disseminated infections with poorly pathogenic mycobacteria), fungal (persistent mucocutaneous candidiasis, invasive aspergillus or mucor), protozoal (cryptococcus meningitis, chronic diarrhea due to giardia) infections Interstitial pneumonitis due to *P.jiroveci*, lymphopenia in infancy (< 2,500/μl)	Full blood count and differential
		Lymphocyte subsets by flowcytometry (CD3, CD 4, CD 8, CD 16 and CD 56, CD 19)
		Serum immunoglobulin levels (IgG, IgA, IgM, IgE)
		T cell proliferation assay
		Delayed type hypersensitivity test (using purified protein derivative, mumps, tetanus vaccines).
		Further tests may be necessary
Antibody deficiency	Recurrent/severe sinopulmonary infections, arthritis, meningitis, osteomyelitis, infections with capsulate bacteria, chronic diarrhea or malabsorption. Viral infection (meningoencephalitis with entero viruses)	Serum immunoglobulin levels (IgG, IgA, IgM, IgE)
		Lymphocyte subsets (including CD 19). Functional antibodies (isohemagglutinins *, anti tetanus/anti diphtheria IgG, anti pneumococcal/anti typhoid Vi IgG**)
		Further tests may be necessary
Other well defined immune deficiencies	Ataxia and telangiectasia (ataxia telangiectasia), cardiac defects, hypocalcemia, hypoplastic thymus and dysmorphic facial features (chromosomal 22q.11.2 deletion), eczema in infancy, recurrent skin abscesses, pneumonia with pneumatoceles and dysmorphic facies (hyper IgE syndrome)	Tests depend on disease
Phagocytic defects	Recurrent skin abscesses or cellulitis, visceral abscesses, mucocutaneous ulceration, granuloma formation, invasive fungal infection,	Full blood count and differential. If neutropenia, identify cause. If neutrophil count normal, depending on clinical features tests for chronic granulomatous disease (nitro blue tetrazolium assay, dihydro rhodamine assay) or leukocyte adhesion defect type 1 (CD18, CD 11 a, CD 11b, CD 11c by flowcytometry) and type 2 (CD 15 by flowcytometry)
	Disseminated mycobacterial disease, BCGosis, disseminated non typhoid salmonellosis (MSMD)	Tests in specialized laboratories
Complement defects	Infections with encapsulated bacteria, recurrent meningococcal infections, lupus like vasculitis	Functional hemolytic complement assays (CH 50 and AP 50)
		If abnormal, is followed by evaluation of individual complement components

*After one year of age.

**After 2 years of age.

Molecular and genetic diagnosis of PID is in its infancy. As of today, molecular and genetic diagnostic facilities are available only for X linked SCID, XLA and recently for chromosomal deletion 22q11.2. Only 17 patients (23.2%) had their diagnosis confirmed by genetic assays, similar to the Indian experience (25%) [35].

Intra venous immune globulin (IVIG) is expensive and out of reach of most patients. In addition, the preparations available are from western sources, and may not reflect the spectrum of microbes present in an eastern setting [34]. All patients with XLA, and patients with CVID < 18 years are on IVIG therapy. This is provided free of charge at government hospitals. Unfortunately, adult patients cannot avail themselves of this facility, and they are treated fresh frozen plasma. Patients with chronic granulomatous disease are on co trimoxazole and anti-fungal prophylaxis, but IFN γ therapy is not offered.

Hematopoietic stem cell transplantation (HSCT) is not available in Sri Lanka. Thus far, one patient, with X linked SCID has been offered this therapy, in India, and is 3 years old at present. Most other children with SCID died in the first year of life. One other patient, with CD 40 deficiency underwent a HSCT in the USA.

Sri Lanka uses the oral polio vaccine (OPV), and coverage is universal. The last case of polio was recorded in 1991. However, patients with common variable immune deficiency may excrete a mutated, potentially dangerous vaccine derived polio virus (iVDPV) for long periods, as was seen with one patient with CVID within the present cohort. This mutated virus may pose a threat to the community as well [39].

The Allergy and Immunology Society of Sri Lanka, set up in 2000, bringing together professionals interested in immunology, with links to international organizations such as WAO, APAAACI and IUIS has slightly changed the dynamics. International collaboration has made the task of PID diagnosis slightly easier.

The present study was confined to passive case detection and the prevalence of PID in the country could not be ascertained. However, most patients suspected of an underlying immune deficiency are referred to this unit. Basic investigations and a few advanced tests are carried out, but more advanced tests are not available. Reference values for the Sri Lankan (or even South Asian) populations are not documented. However, with these limitations, the present study is one of the few studies in the South Asian region, and the first outside India to provide an overview of PID.

## Conclusion

The spectrum of PID is similar to that described in the west, with antibody deficiency (mainly CVID and XLA) the most common. However, IgA deficiency is uncommon. Treatment of antibody deficiency has improved life expectancy and quality of life in most patients. However, early diagnosis of adult patients, and treatment of cellular immune deficiency is inadequate. Much needs to be done to educate clinicians and provide better diagnostic facilities for PID.

## Competing interests

The authors declare that they have no competing interests.

## Authors’ contributions

NRDS–Concept and design of study, acquisition of data, analysis of data, writing of manuscript. SG–Acquisition of data, including immunoassays. DR–Acquisition of data, including immunoassays. GDW–Acquisition of data, including immunoassays. All authors read and approved the final manuscript.

## Authors’ information

NRDS MBBS, MD, Consultant Immunologist, SG MBBS, MD, Consultant Immunologist. DR MBBS, Medical Officer. GDW MBBS, Medical Officer.
